# Optical Fiber Networks for Remote Fiber Optic Sensors

**DOI:** 10.3390/s120403929

**Published:** 2012-03-26

**Authors:** Montserrat Fernandez-Vallejo, Manuel Lopez-Amo

**Affiliations:** Department of Electric and Electronic Engineering, Public University of Navarra, Pamplona 31006, Spain; E-Mail: mla@unavarra.es

**Keywords:** Raman amplification, fiber-optic sensor multiplexing, remote sensing, fiber Bragg Gratings (FBGs)

## Abstract

This paper presents an overview of optical fiber sensor networks for remote sensing. Firstly, the state of the art of remote fiber sensor systems has been considered. We have summarized the great evolution of these systems in recent years; this progress confirms that fiber-optic remote sensing is a promising technology with a wide field of practical applications. Afterwards, the most representative remote fiber-optic sensor systems are briefly explained, discussing their schemes, challenges, pros and cons. Finally, a synopsis of the main factors to take into consideration in the design of a remote sensor system is gathered.

## 1. Introduction

A sensor network is an array of sensors that are deployed either directly inside the element to be assessed or very close to it. Optical fiber sensor networks represent a significant improvement over traditional sensors networks ensuring a wide range of application areas such as environmental, safety and security monitoring. As regards the links, fiber-optic sensors can be interconnected by wireless, copper wire or optical fiber [1]. Among them, the last one is the chosen technology.

The crucial feature of optical fiber is its dual functionality, it is not only a sensing structure in view of the measurand induced changes of the light properties that propagates in the fiber, but also a communication channel, meaning there is no need for an additional telemetry path, contrary to what happens in all other sensing technologies [2]. Other key advantages are: almost total immunity to external electromagnetic interference, multi-path reflections from natural and man-made objects and interruptions by bad weather; on top of that, it exhibits vast bandwidths and low transmission losses, enabling very wide geographical coverage; intrusive data interception is more difficult when using dielectric optical waveguide transmission media than free-space radio propagation or metallic wires; where necessary, fiber interconnects allow the sensors to be inserted within the structures being monitored, they can operate without electrical powering of local batteries outside the terminal nodes and they reduce the risk of sparking in combustible environments [3].

Consequently, fiber-optical sensor networks have emerged as a powerful tool for condition assessment of the system under consideration [2]. They have found a promising niche in the field of Structural Health Monitoring (SHM) which refers to *the use of in situ, continuous or regular measurement and analyses of key structural and environmental parameters under operating conditions, for the purpose of warning impending abnormal states or accidents at an early stage to avoid causalities as well as giving maintenance and rehabilitation advice* [4]. Fiber-optic sensor networks provide sensing solutions for almost all kind of applications and environments: from large scale structures, including bridges and other civil constructions to large natural environments [5].

Despite their marked advantages, optical fiber networks for sensors pose three challenges. The first is the need to increase the number of sensors that can be multiplexed on a single network while ensuring good signal quality. The most fundamental motivation for multiplexing fiber optic sensors is the cost, due to the fact that if the optoelectronic unit, which is the most expensive device in the network, is shared among a high number of sensing points the cost per sensing element decreases [6]. Wavelength division multiplexing is one of the best methods of multiplexing as it uses optical power efficiently, and also, it can be easily combined with other multiplexing methods, allowing a large number of sensors in a single fiber line [7]. The second demand is to ensure service continuity in the event of point failure(s) on the network. Resilience or self-healing is the ability of continue operating despite one or more points of failure on the network, which will be the key issue for practical FBG sensor systems [8]. The continued operation of the sensor network after accidental or malicious damage is of increasing importance when the structure being monitored is of high value (oil pipelines, power transmission lines, *etc.*); human safety is at risk (bridges, dams, chemical storage sites, nuclear plants, *etc.*) or perimeter security is a concern (airports, banks, *etc.*) [4]. The last one is to enable the possibility of remote sensing. The first two challenges are common to all optical fiber networks, while the last one, it is more specific and it is thoroughly discussed below.

Remote sensing using optical fiber systems has received an increasing attention in recent years due to the fact that it has proven to be a useful tool for monitoring a wide range of parameters in many fields. In general, the pivotal idea behind remote sensing concept is the continuous monitoring of structures from a central station located tens or hundreds of kilometers away from the field through the critical location of sensors, which send information to the central station, without the necessity of electrical power feeds in the remote locations. This remote capability allows immediate damage detection so that necessary actions can be quickly taken. Furthermore, this strategy removes the logistical inconvenience of electrical power feeds to remote locations [9,10].

More powerful remote sensing systems can find important applications in structural monitoring of large infrastructure components, such as oil or gas pipelines, ultralong bridges and tunnels, river banks and offshore platforms [9,11]. There are other promising applications of remote sensing to be highlighted. Firstly, tsunami detection and warning before their arrival to the coast, which is intended to mitigate as far as possible the disasters [12,13]; secondly, geodynamical monitoring such as surveillance of volcanic and tectonic areas which is used to predict the possible evolution towards critical stages or to detect landslides [14]; and finally, railway applications like train speed measurement, derailment, wheel defects and rail crack detection, to name but a few. Methods currently in use suffer from complexity and slow response times [15]. The methods of tsunamis detection gives an example of how of cumbersome the present techniques are: a pressure sensor, which needs electrical supply, is located in the bottom of the sea, this sensor sends acoustic signals to the buoy in the surface and it transmits the data to the satellite which launches it to the surveillance centers. Optical fiber systems, nevertheless, are secure and faster, offering very high accuracy as well as the possibility of real time measurement. These potential practical applications are the justification of this growing interest.

A criterion of classification optical fiber sensors (OFS) is according to the spatial distribution of the measurand, so, they can fit into two classes: distributed OFS, for example, those based on Brillouin scattering or Raman scattering; and discrete sensors such as fibre Bragg gratings (FBG) or Fabry-Perot.

Distributed optical fiber sensors are attracting more and more interest thanks to their wide range of potential industrial applications in strategic sectors such as energy, security, defense and transportation, among others. In particular, fiber sensors based on Brillouin optical time-domain analysis (BOTDA) exploit the dependence of the Brillouin frequency shift parameter on strain and temperature [2,16–18]. These distributed Brillouin sensors are able to measure with highly accurate over long single mode fibers exceeding several tens of kilometers. This fact allows monitoring civil structures to which optical fibers can be attached with highly accurate distributed measurements over long single-mode fiber. Nevertheless, the measurement range of these systems has a trade-off between the spatial resolution and the measurement range. For this reason, current research in BOTDA sensors has two different scopes: firstly, high-resolution sensors with centimeters spatial resolution, but for relatively short-distances fibres and, secondly, long-range BOTDA sensors, which are able to perform measurements in tens of kilometre fibres with metre resolutions. In addition, if these schemes were for remote sensing, this measurement range should be divided by a two factor, because of the fact that stimulated Brillouin scattering only occurs when incident pump light is contradirectional to the signal. For this reason, some recent published systems, whose length is 100 or 120 km [19–22], would reach 50 km or 60 km in a remote sensor system. This effective range limits their use in certain applications in which the distance to monitor is larger. We consider that a detailed discussion of this promising field goes beyond the scope of this review.

Regarding Fabry-Perot sensors [23–25], they have been developed for a variety of applications. In fact, they can provide high sensitivity, dynamic range, and response speed for measurement of temperature, strain, pressure, displacement and magnetic field. However, their development has not been as quickly as some had expected a decade ago because weak interferometric signal, costly signal processing and difficulty in wavelength division multiplexing are two major intrinsic drawbacks, limiting their applications considerably. Advances in technology and the availability of commercial Fabry-Perot sensor products will make them cost competitive in an increasing number of fields. They have some areas where rapid growth is expected during next decade: optical networks at large, and in particular, biomedical applications, smart military and commercial structures, industrial equipment monitoring, and in the oil and gas industry [26]. Consequently, few studies have been published on fiber optic networks for remote sensing including Fabry-Perot sensors, one of them reaches 50 km [27,28].

Among the wide variety of available sensors, both optical and non-optical, Fiber Bragg Gratings (FBGs) are the strongest candidates for this kind of systems due to the numerous advantages they offer. They present roughness in hostile environments, good linearity, simple demodulation concepts, electromagnetic immunity, compactness, embedding capability, commercial availability, small size and low cost. On top of that, one of the major advantages can be attributed to their wavelength-encoded information, thus the information remains immune to power fluctuations along the optical path. Another attractive benefit is their high multiplexing capability. These inherent characteristics make them attractive for applications in harsh environments and smart structures [29,30]. But, their cross-sensitivity effect, Bragg wavelength shift is simultaneously sensitive to both strain and temperature, supposes a great handicap in real engineering applications. Thereby, some methods are needed to discriminate both measurements [31,32].

Two additional important issues must be taken into consideration when fiber-optic remote sensing systems are designed. Firstly, the interrogation system, and secondly, the most suitable amplification method must be chosen to compensate for the losses undergone by the light.

As far as interrogation systems are concerned, they must allow obtaining the Bragg wavelength shift when a physical measurand acts on the grating. Consequently the determination of the Bragg wavelength allows quantifying the state of a particular parameter. The simplest method is using conventional spectrometers which are widely used in laboratories. However, high precision optical spectrum analyzers are unsuitable for real sensor systems, not only because of their high cost and large-sized but also because their slow scanning speed limits dynamic sensing [33]. Interrogation techniques must provide high sensitivity to Bragg wavelengths shifts, large measurement ranges, immunity to optical power fluctuations, low environmental sensitivity, extension to sensor multiplexing, simplicity and low cost [34]. Different methods have been reported for measuring the wavelength-encoded temperature or pressure changes of FBG. But probably, the sensor interrogation system with the greatest commercial success is based on a wavelength swept laser, which scans the reflection spectra of the Bragg gratins using the power detected by a photodiode. However, this type of systems is inherently limited in its reach due to Rayleigh scattering noise [10]. A matter of fact, the choice of the interrogation method depends upon several factors like type and range to be measured, accuracy and sensitivity required, number of sensors being interrogated and cost of the instrumentation. In conclusion, there is no a perfect interrogation method suitable for all the applications, but there is enough flexibility in order to reach an optimum interrogation method for any application [5,29,31,33,34]. By way of example, in the case of the measurement frequency is of prime importance, FBG interrogation methods can be classified as in reference [35]. Some cases of bridge monitoring where high frequency is needed to capture strain variations due to passage of speeding trucks can be found in the literature. To give an example, reference [36] is able to reach a data acquisition up to values of 500 Hz, thus, the assessment of the structural behaviour under current traffic conditions can be performed. In special, they show the results obtained during the passage of vehicles on normal traffic conditions, trucks circulation at an average speed of 50 km/h, logged with an acquisition rate of 200 Hz. As a matter of fact, recently some preliminary studies on the use of FBG sensors for monitoring railway footprints of high-speed trains, passing at speeds between 200 and 300 km/h, in the Madrid–Barcelona (Spain) high-speed line, are being developed with a FBG sensors interrogation system which allows the simultaneous monitoring of fours sensors at 8,000 samples/s [37].

The methods proposed and reported in the scientific literature, with the essential motivation of extending the distance while maintaining a good signal to noise ratio, usually include optical amplification to compensate the losses [38]. The first systems were based on broadband light sources in which case the distance was limited to a maximum of 25 km, mainly due to Rayleigh Scattering [39]. In order to surpass this limit, FBG sensors systems based on a fiber linear laser scheme are a promising alternative due to the fact that they have demonstrated several advantages, such as high resolution or high signal-to-noise ratio (SNR) against noise environments in practical applications. These systems usually include Raman amplification [40], or Raman amplification merged with other kinds of amplification: Brillouin, Erbium doped fiber or both [41–43]. To the best of our knowledge the longest distance covered by a FBG sensor system for a single FBG reported to date reached 253 km, the displacement sensor system based on a fiber loop mirror and a long period grating inside of the loop mirror is interrogated by a commercial optical time-domain reflectometer (OTDR) [44]. Following these approaches this research field is being extensively investigated at present.

Distributed Raman amplification (DRA) is the most utilized optical amplification in remote sensing networks. The systems profit from the main benefits of the Raman amplification. Firstly, it is a process inherent to germane-silicate fibers. This is a great advantage because, as no special fiber is needed to obtain Raman amplification, the transmission fiber acts as an amplifier itself. Thus, it makes the amplifier configuration very simple. Secondly, gain may be achieved at any signal wavelength simply choosing an appropriate pump wavelength. As a consequence of this second advantage, the amplification wavelength range may be extended and the ripples in the spectral gain may be reduced by using multiple pumps at different wavelengths and adjusting carefully their powers. These properties are extremely useful for the development of broadband multichannel systems, in which a large number of signals are multiplexed. Finally, as DRA name suggests, it has the capability to provide distributed amplification [45–47].

DRA is able to overcome the losses at every point along the transmission fiber, or in other words, it utilizes the transmission fiber in the network as the Raman gain medium to obtain amplification. Moreover, its use also encloses two crucial assets: improving the signal to noise ratio and reducing the nonlinear penalty due to the fact that the overall excursion that the signal experienced is reduced. The signal is prevented from decaying and increasing as much as it would make if the amplification were discrete. This improved noise performance may be used in different ways: extending the reach between repeaters, improving the transmission capacity, or the most interesting for remote sensing, expanding the total reach of the transmission system. There is, even, a forth possibility, the margin released can be used for decreasing the signal power injected in order to postpone the onset of nonlinearities or reduce their effects [48].

Although distributed Raman amplification is widely included in the remote sensor network due to its attractive advantages, it also entails some limitations which hamper the reach [45]. Two of the most severe challenges to overcome are, firstly, Rayleigh reflections and, secondly, nonlinear effects that may be enhanced in distributed Raman amplifiers since the path average signal power is higher along the transmission fiber when compared with unpumped fiber. In addition, when pump and signal are copropagating the cross-coupling between the pump and the signal also must be considered [45]. Among them, the multipath interference (MPI), primarily attributed to double-Rayleigh scattering (DRB) along the length of the transmission fiber, is the most important obstacle when the reach of the transmission system wants to be expanded [49,50]. It occurs in an optical fiber due to small inhomogeneities or microscopic variations in the refractive index [51]. An intuitive if not rigorous explanation of double Rayleigh scattering is as follows: light is scattered randomly in all directions and a small portion of the signal reflects back due Rayleigh backscattering, this reflection is amplified by the Raman gain and reflected once again due to Rayleigh backscattering and it can be recoupled into the forward direction. Therefore, the double Rayleigh scattering is amplified twice and it scales as the length since the gain is accumulated over tens or hundreds of kilometers. In summary, DRB produces a large number of low power replicas of the signal with random delays and phases that propagate along with the signal. The problem associated with this extra noise is due to its inherent characteristic of occupying the same spectral region as the signal and consequently is not easily distinguish it from the real signal. So far electrical measurements have been preferred for characterization of DRB [52]. The use of multiple gain stages optically isolated one from another is a well-known technique of reducing the DRS, but usually when distributed amplification is used is not possible to include [53]. Another way to limit the DRS is to combine the Raman amplifier with and EDFA to form a hybrid amplifier [51].

Table 1 summarizes the state of the art of remote sensing systems for optical fiber sensors in chronological order taking into account the most representative characteristics of the systems. When the first fiber-optic sensor networks were designed with some kilometers [54,55], they were considered as remote sensor systems.

However, nowadays fiber-optic sensor systems can cover hundreds of kilometers. Because of this, the summary table only contemplates remote sensor systems with, at least, some tens of kilometers.

## 2. Demonstrated Systems

This section is devoted to explain more carefully the most representative remote fiber-optic sensor systems for fiber optic sensors presented in Table 1, discussing their schemes, pros and cons. They are going to be evaluated not in chronological order as in the list, but taking into account the system length.

### 2.1. 22 km System

Diaz *et al.* demonstrated in references [57,58] some networks based on bus topology for wavelength division multiplexing of optical sensors using distributed fiber Raman amplification in order to compensate for the inherent losses of the transmission channel, the optical fiber, and the passive devices.

**Figure 1.**
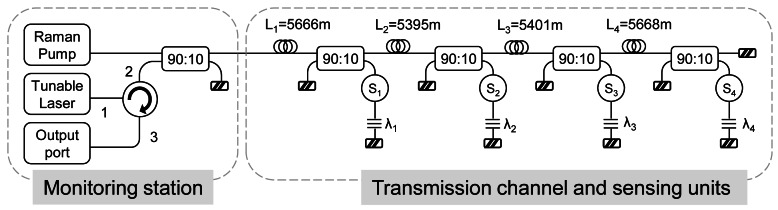
Wavelength-division-multiplexed distributed fiber Raman amplifier bus network. Adapted from [57,58].

The bus consisted of a spine section that connects a series of directional couplers which lead the signal to the sensing elements, followed by the fiber Bragg gratings (FBGs), as Figure 1 shows. In this case, approximately 5 km SMF fiber spans were placed between the couplers. However, there is no strict constraint on the lengths. The sensors can provide amplitude, phase, or polarization modulation in response to the chosen environmental influence, while the FBGs reflect incident signals and therefore uniquely identify the sensors being addressed.

It is noteworthy that bus architecture is one of the most widely used, mainly owing to its simple cabling requirements when compared with star networks, and its potential to increase the degree of integration of different sensors [73]. Moreover, it gives the opportunity of both combining different kind of sensor in a network and distributing the sensors at critical points. But, this topology has to overcome two main problems: the necessary minimum detectable power at the receiver and the dissimilar power received from each sensor due to the different number of couplers each signal finds on its optical path. As can be observed in the results of reference [57,58], perfect equalization of all channels is a difficult task.

### 2.2. 50 km Systems

**Figure 2.**
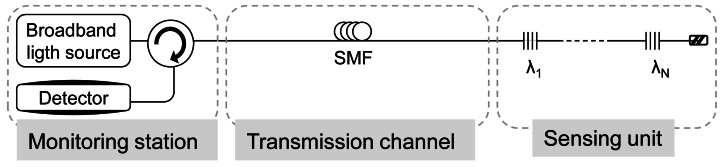
Conventional FBG sensor system.

Conventional sensing systems use a broadband light source as a sensing probe signal, it is shown in Figure 2. In these cases, when the sensing system length is more than 25 km it is difficult to detect the sensing signals because of Rayleigh scattering-induced optical noise as well as loss of background signal in the transmission fiber [40]. To enhance the performance of sensing systems, a fiber laser-based sensing probe with a narrow bandwidth and a high extinction ratio is a possibility to consider. These systems require the two key elements of a basic laser scheme: a gain material that provides amplification and an optical cavity that traps the light, creating a positive feedback. FBGs are used mostly for both sensing function and selection of the wavelength. Furthermore, the utilization of an amplifying medium between the gratings and the mirror pumped inside or outside the cavity provides gain and thus the lasing occurs when the total gain in the cavity overcomes the total cavity loss.

**Figure 3.**
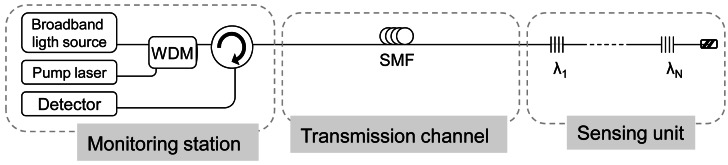
Conventional FBG sensor system with distributed Raman amplification. Adapted from [40].

Nakajima *et al.* [40] introduced, for the first time, distributed Raman amplification into the simplest conventional FBG sensor system which is composed by a broadband light source, optical circulator, FBGs multiplexed in series and a detector. Figure 3 depicted the used scheme. The limited transmission distance of the conventional systems was overcome and the new system achieved to interrogate a FBG as far as 50 km with a SNR of 15 dB.

**Figure 4.**
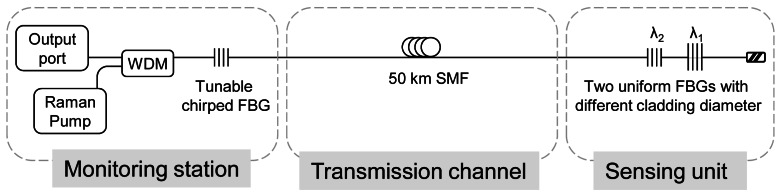
Experimental setup for the multiwavelength Raman fiber laser based on FBG located in serial configuration for a long distance remote-sensor system. Adapted from [39].

Han *et al.* [39,59] proposed two remote sensor systems based on a linear cavity Raman laser configuration formed by different FBGs to create the cavities. In the first approach, a tuneable chirped FBG and two uniform FBGs with different diameter were used, Figure 4 depicted it. In the second one, a tuneable chirped and a multiple phase-shifted FBGs. As mentioned before, the FBGs are used for both sensing function and selection of the wavelength. Both systems achieve good optical signal-to-noise ratio (OSNR), approximately of 50 dB, but increasing the number of multiplexed FBG is an awkward task. The fact of locating the FBGs in serial configuration hampers the expansion of the network because it is a complicated task to adjust correctly the cavity losses at each wavelength to achieve oscillation in all the desired channels.

Peng *et al.* [60] also suggested similar systems using Erbium doped waveguide amplifier (EDWA) and SOA. It uses a fiber loop mirror and three FBGs to create the cavities. Although the OSNR is about 50 dB, the achieved length is shorter, 25 km, limited mainly by the SOA.

Rao *et al.* [11] presented a tunable fiber ring laser with hybrid Raman-Erbium doped fiber amplification with a star/bus configuration. Despite the scheme is more complicated than others, the results show an excellent OSNR of 60 dB.

**Figure 5.**
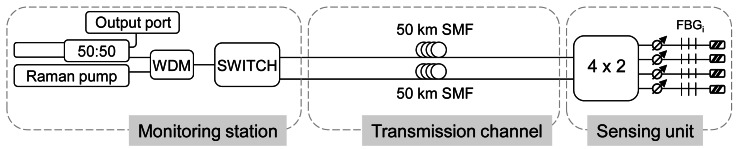
Experimental set-up for the multiwavelength Raman fiber laser for long distance remote-sensing with self-healing beaviour. Adapted from [66].

Finally, we developed a long distance FBG sensor system [66], whose scheme is depicted in Figure 5. One again, the long-distance remote sensing system is based upon a multiwavelength Raman laser which offers an OSNR of 46 dB. As it has been explained, most of the systems proposed in the literature usually have the FBGs located in a serial configuration. The demonstrated system was an improved version of [40] in some aspects. Firstly, the FBGs are disposed in parallel in the sensing extreme. Thereby, power instabilities are diminished through achieving easily power equalization for all the channels and to enable an easy repair of the sensors when needed or exchange the kind of sensor depending on the needs. However, the price to pay is a much higher Raman pump power. Secondly, we would like to stress that the system was designed to be inherently resilient to fiber failures. Resilience or self-healing is the ability of continue operating despite one or more points of failure on the network, which will be the key issue for practical FBG sensor systems [8,73]. The merge of concepts, resilience and remote sensing, is still an open field of research with few references in the literature, but with a promising future.

The self-healing beaviour was achieved with the 1×2 switch which performs the necessary selection of launch point. With this configuration, the system uses “shared protection” to re-establish service after a failure. In normal operation the switch is connected to the “working fiber” but when a failure occurs, it is switched to the “protection fiber” (the other 50 km of SMF places in parallel) [74]. Consequently, only the working fiber is used in normal operation and the protection fiber is activated in the event of a failure.

To sum up, these systems based on laser configuration have the asset of removing the requirement of an additional broadband light source and significantly improves the sensing signal quality.

### 2.3. 100 km Systems

**Figure 6.**
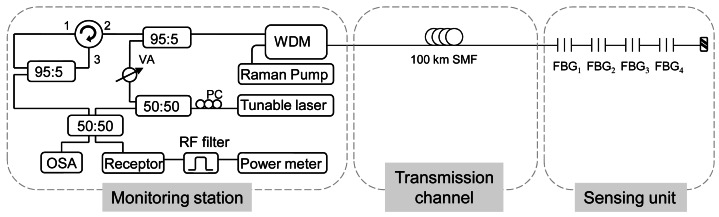
Experimental set-up for the FBG interrogation technique for remote sensing. Adapted from [68].

In [68], we demonstrated the feasibility of a novel Fiber Bragg Grating interrogation technique for remote sensing based on the use of a hybrid Raman-Brillouin fiber laser configuration. It is displayed in Figure 6. The laser comprised 100 km of standard single-mode fiber (SMF) in a linear cavity with four Fiber Bragg Gratings (FBGs) arranged in series. The FBGs were used for both sensing function and selection of the lasing wavelengths.

The operation mode of the interrogation system was as follows. The Raman pump laser provides enough gain in the cavity so that the system is set just below the lasing threshold. Then the tunable laser, which provides the Brillouin gain, makes a sweep in wavelength. When it is sweeping in wavelength, there are two possible situations:

- Firstly, when the wavelength of the tunable laser matches with the reflection band of one of the FBGs. Then the hybrid Raman-Brillouin gain is enough so the laser action takes place. The laser signal wavelength is separated from the tunable laser wavelength by the Brillouin frequency shift in the fiber;
- Otherwise, when the tunable laser wavelength is outside the FBGs band. There is just Rayleigh reflection of the laser and some spontaneous Brillouin scattering, but there is no laser signal.

This detection system overcomes the problem of low signal to noise ratio due to Rayleigh noise which usually limits the wavelength-swept laser interrogation system. With it, Rayleigh noise is relegated to low frequencies (around DC) in the detected electrical signal, whereas the fiber laser signal is transferred to noise-free RF frequencies around the Brillouin frequency shift in the fiber.

This long distance FBG sensor system had some advantages over some previously reported ones in the literature. First, the obtained signal to noise ratio of 30 dB was much greater than in the traditional system of interrogation, where the main constraint is imposed by Rayleigh scattering. Secondly, the Raman pump power used, 0.77 W, was lower than in systems where the length achieved is 50 km. Hence, our system faced some disadvantages of the long-distance sensor systems solely based on multiwavelength Raman lasers. For example, the serial topology could seem an obstacle to achieve power equalization for the channels, however, in this scheme it is not a problem, because although the system is based on a long distance laser structure, the channels must not lase at the same time, wherein the mode competition has not crucial influence. Therefore, the system is specially suited for the usual FGBs sensors arrays.

In conclusion, with the proposed system the sensor signal is detected in the radio frequency domain instead of the optical domain so as to avoid signal to noise ratio limitations produced by Rayleigh scattering. Experimental results demonstrate that the Bragg wavelength shift of the FBG sensors can be precisely measured with good signal to noise ratio when the FBG are used for temperature sensing. Despite the complex set-up, the experiment results prove that this method has advantages of high signal to noise ratio, remote sensing and immunity against the light source power fluctuations.

**Figure 7.**
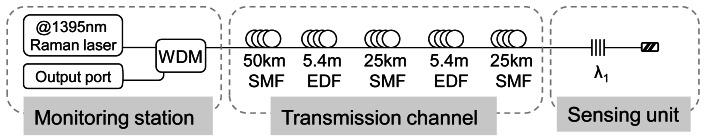
Experimental set-up for the FBG sensor system. Adapted from [27].

Hu *et al.* [27] experimentally demonstrated another 100-km long distance FBG sensor system based on erbium-doped fiber and Raman amplification as Figure 7 shows. The reflected Bragg wavelength spectrum achieved an OSNR of 30 dB with 1 W power at 1,395 nm. The working principle is based on the generation of a first order SRS around 1,480 nm by the pump power at 1,380 nm, this peak at 1,480 nm has enough power to act as pump of the EDF sections allocated along the transmission channel. Thus, the area around 1,350–1,570 is amplified thanks to EDFA.

When monitoring stations of reference [27] and reference [59] are compared, it is obvious that reference [27] is characterized by its simplicity. However, multiplexing several FBG sensors with this system would not be a simple task because the final amplification of the system is based on EDFA whose gain profile is non-flat. This intrinsic feature of EDFA would hamper the equalization of the FBG sensor signals, especially, when the FBGs are located in serial configuration and the individual loss control for each wavelength is not possible. As has been explained, reference [59] solved this problem and it was able to multiplex several FBGs without any problem.

### 2.4. 120 km System

**Figure 8.**
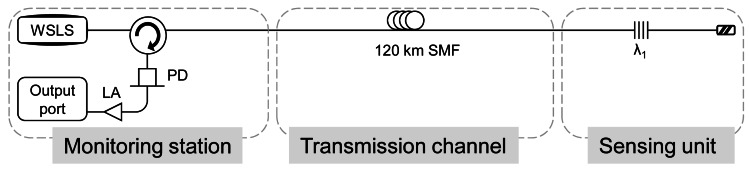
Experimental set-up for the FBG sensor system. PD: photodetector. L.A.: logarithmic amplifier. WSLS: wavelength-swept light source. Adapted from [62].

Saito *et al.* developed in reference [62] an ultra-long-distance (120 km) FBG sensor system using a wavelength-swept light source with output power turned ON-OFF and timing synchronized to the sweep signal to reduce optical noise caused by Rayleigh scattering generated from the transmission fiber. This method does not need an optical amplifiers or pumping laser and copes with the limiting factor of Rayleigh scattering. The corresponding scheme is displayed in Figure 8.

A conventional wavelength-swept light source has a wavelength sweep time of several hundred milliseconds in the C-band range. Thus, the photodetector detects the FBG reflection spectrum when the wavelength-swept light source is sweeping the wavelength. At this time, Rayleigh scattered is simultaneously detected by the photodetector, so the final SNR is restricted by it. In order to overcome this issue, the authors developed a high-speed wavelength-swept light source. The FBG sensor system using the wavelength-swept light source can monitor the FBG reflection spectrum after wavelength sweeping is finished, because the wavelength-swept light sources sweeps the wavelength quickly. If the output power of the wavelength-swept light source can be turned OFF immediately after the wavelength sweep has finished, Rayleigh scattered light detected by the photodetector when the FBG spectrum is observed by the photodetector can be considerably reduced.

For the first time, pulsed waves were employed to interrogate FBG placed far away from the monitoring station. Despite the fact that this kind of systems is composed by expensive devices which increase the total cost, systems based on pulsed regime can be an appealing option in order to avoid building up Rayleigh scattering along the long transmission channel.

### 2.5. 155 km System

**Figure 9.**
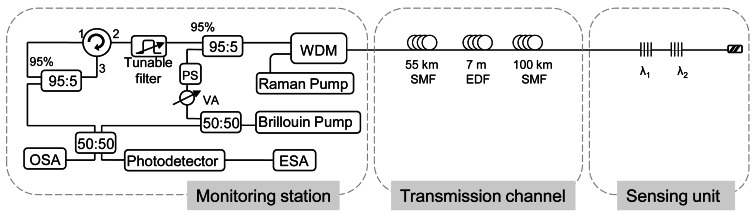
Experimental set-up sued for remote interrogation for two FBGs. Adapted from [43].

This remote FBG interrogation technique combining Raman, Brillouin and Erbium gain in a fiber laser is an improved version of the previously exposed system in Section 2.3 and whose design is presented in Figure 9 [43]. The enhanced method was able to interrogate two FBGs located in series at 155 km away from the processing unit using a pump power as low as 0.6 W. The heterodyne detection, once more, overcame the problem of low SNR due to Rayleigh noise which usually limits the wavelength-swept laser interrogation system and brings forth a signal to noise ratio of approximately 10 dB.

In this system, Raman, Brillouin and EDF amplifications are combining. The Brillouin laser pumps the channel, giving rise to an active medium whose gain is the combination of Raman an Erbium gain profiles. The power launched into the channel has to be enough to induce Raman amplification and to pump the 7 m of highly EDF. The EDF was inserted after 55 km in order to optimize the combined amplification effect.

Hybrid Raman/erbium doped fiber amplifiers are a promising technology for future dense wavelength-division-multiplexing (DWDM) systems [75]. These amplifiers are designed in order to maximize the span length and/or minimize the impairments of fiber nonlinearities, and to enhance the bandwidth of EDF. In the proposed system, the hybrid amplifier offers a maximum gain with the combination of the EDFA and Raman amplifier gain and reduces the effects of Rayleigh scattering. However, the insertion of an EDF piece in the transmission channel can increase its complexity.

Hu *et al.* in reference [63] also showed a 150 km long distance FBG sensor system combining Raman and EDF amplification. The FBG 150 km far away from the monitoring station only offered 1 dB of OSNR. However, this low OSNR did not comply with the minimum requirements from a practical point of view.

### 2.6. 200 km System

**Figure 10.**
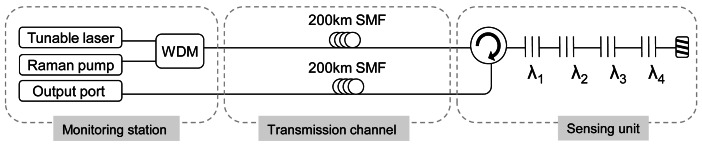
Schematic depiction of the ultra-long fiber Bragg grating sensor system [69].

We demonstrated a 200 km remote FBG multiplexing system which is schematically depicted in Figure 10. At first glance, it is noted that the scheme is remarkably simple. Not only its design, but also its operation mode is straightforward because it is based upon a wavelength swept laser (with a bandwidth of 100 MHz) to scan the reflection spectra of the FBGs. The fundamental difference of reference [69] compared to the rest set-ups to date lies in the transmission channel which consists of two identical optical paths. The first path intended to launch the amplified laser signal by means of Raman distributed amplification, while the other one is employed to guide the reflection signal to the monitoring station.

The justification of using two paths, which doubles the needed fiber in the system, is based on the reduction of the effective cost of fiber optic components, especially SMF cables. Furthermore, in real applications the final cost of the installed system is not significantly increased if two fibers are used instead of only one into a cable.

It is outstanding that the design system was a low noise configuration because it coped with the two principal dominating sources of noise of a fiber Raman amplifier. The amplified spontaneous emission (ASE) generated by spontaneous Raman scattering is addressed by the FBGs, since they only reflect the Bragg wavelength. And on top of that, the multipath interference (MPI) noise mainly produced by Rayleigh backscattering (RB) does not play a crucial role because the reflected signal travelled through a different optical path than the launched tunable laser and the distributed Raman amplification.

As mentioned above, how to place the sensors is also an issue to take into account. In this case, the FBGs are disposed in serial configurations. Initially, it could seem an obstacle to achieve power equalization for the channels, however, in this scheme it is not a problem, because the system is not based on a long distance laser structure. In the proposed system, the channels equalization depends on both the non-uniform shape of the Raman profile and the insertion loss of the FBGs located in front of the sensor interrogated in each moment. Specifically, the optical signal to noise ratios from the four FBG remotely multiplexed is approximately 20 dB when 0.72 W of Raman pump laser and 10.68 dBm of tunable laser are launched into the system.

In conclusion, reference [69] showed an ultra-long FBG sensor system which addresses the most limiting factor of remote sensing: Rayleigh Backscattering. Over and above, it accomplished it in a simple-way.

### 2.7. 230 km System

Saitoh *et al.* proposed in reference [10] an improved adaptation of its ultra-long-distance (120 km) FBG sensor system above explained in Section 2.4 [62]. The operation mode have been explained, but in summary, the FBG sensor system can observe the FBG reflection spectrum after wavelength sweeping has finished, because the output power of the high-speed pulse-driven swept light source is turned off immediately after wavelength sweeping finishes.

In reference [62], there was not any amplification. However, in this enhanced version the light output of the HSLS is amplified by an EDFA in order to observe a SNR of 4 dB even when the FBG is connected using a 230 km transmission fiber.

Although in reference [10,62] only one FBG was connected to the FBG sensor system, others FBGs could be simultaneously connected achieving a multiplexing remote sensing network.

### 2.8. 250 km System

In Section 2.6 has been discussed a low noise remote sensing system wherein the background noise is limited by the noise imposed by the measurement device and the obtained OSNR was 20 dB when the sensor unit was 200 km away from the monitoring station. This great signal to noise ratio encourages trying to reach further distances with the same scheme displayed in Figure 10.

**Figure 12.**
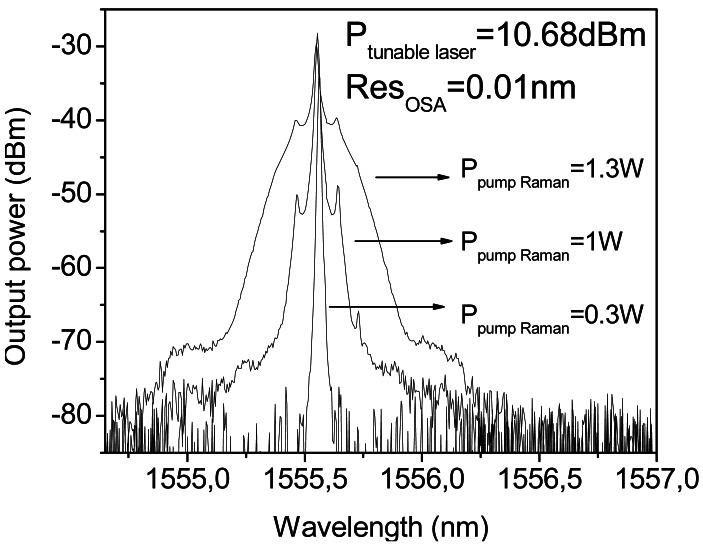
Spectrum of the tunable laser after 250 km.

**Figure 13.**
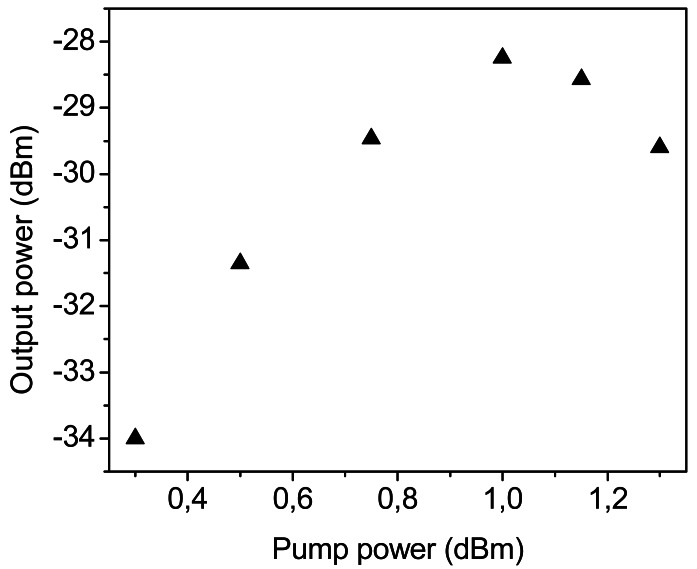
Evolution of laser power *vs.* Raman pump laser.

It is obvious that a higher amount of Raman pump power is necessary since the amount of losses to compensate with the distributed Raman amplification is also higher than in the previous system. To this end, the Raman pump power was increased, but unsurprisingly Brillouin scattering arised, which hampered the signal amplification, as in many fiber communication systems [76]. Figure 12 shows the spectrum of the tunable laser after 250 km length of SMF, it illustrates the progression of the Stokes lines: the higher the pump power, the greater the Stokes lines power and spectrum broadening is also observed. For conventional fibers, the threshold power for this process is a few mW, however, the impairments start when the amplitude of the scattered wave is comparable to the signal power. The biggest problem appears in this kind of situations when the backscattered light experiences gain from the forward-propagating signal which leads to depletion of the signal power. In consequence, there is a practical limitation of the maximum possible gain, as shown in Figure 13.

**Figure 14.**
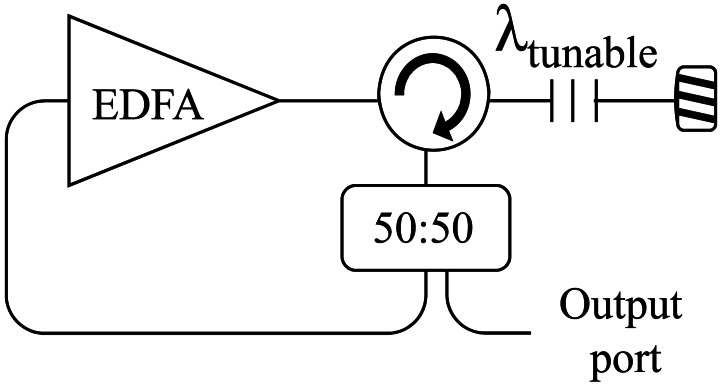
Basic design of the tunable laser.

For lasers with linewidths Δλ much larger than 20 MHz, SBS gain is inversely proportional to Δλ[77]. Thus, for this 250 km length span, a tunable laser, with a wider bandwidth to reduce the problems caused by SBS has been developed. It is based on a previously published scheme by the authors [78] and its scheme is shown in Figure 14. Its bandwidth is 0.6 nm, and it launches 11 dBm with and extinction ratio of 65 dB. The optical signal to noise ratio is 6 dB in the worst case and 8 dB in the best one. This OSNR could be improved using the OSA option sweep high sensitivity because the system is restricted by the noise level imposed by the detection scheme. In this situation, the measured OSNR could achieve 18 dB.

To the best of our knowledge, this ultra-long range fiber Bragg grating (FBG) sensor system is the longest reported system able to multiplex several FBG sensors.

**Figure 15.**
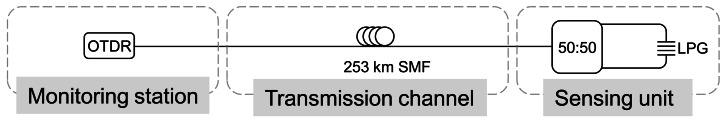
Experimental set-up for the LPG-FLM sensor system. Adapted from [44].

Bravo *et al.* demonstrated an ultralong 253 km remote sensor system based on a fiber loop mirror interrogated by a commercial optical time-domain reflectometer (OTDR) [44]. The experimental setup, depicted in Figure 15, included a fiber loop mirror (FLM) combined with a long period grating (LPG). The high reflectivity of the fiber loop mirror together with the LPG allowed easy detection of displacement by using an OTDR as the interrogation unit. LPGs have been also used as strain, temperature, and refractive index sensors. The high reflectivity of the fiber loop mirror was used as a pulse reflector, and this pulse can be easily observed at 253 km away without any amplification.

The main advantage of this ultralong remote sensor system is the reached length without any optical amplification, but its practical applicability is limited by its low multiplexing capability.

## 3. Results and Discussion

In the course of the previous section, the key parameters in the design of long-distance sensor systems for remote sensing have been discussed. In this section, we want to gather briefly the most important points.

The first consideration is about amplification. When the transmission channel is composed by a span of hundreds of kilometers, if the modulated signal by the sensors wants to be detected, the accumulated losses must be compensated. The most widespread way is the addition of distributed Raman amplification [39,40,57–59,61,65,66,69] due to its advantages. Other possibility is the used of hybrid amplifiers such as the combination of Raman with Brillouin amplification [68] or Raman with EDF amplification [11,42]. Whatever the utilized amplification, its inherent noises and other awkward effects must be counterbalanced. Only a few systems have achieved long distance without amplification [44,62].

Secondly, we have to consider the network topologies. The most common are star and bus. The first one usually uses postamplifiers while bus topology employs distributed amplification. In particular, bus architecture is one of the most widely used, mainly owing to its simple cabling requirements when compared with star networks, and its potential to increase the degree of integration of different sensors [6]. An improved version of the single bus is the double bus or ladder structure. It is composed by two different fibers: the first one is used to send the optical signal towards the sensors, and the second one collects the signal from the sensors. This enhanced topology improves the signal to noise ratio and offers the possibility of multiplexing hundreds of sensors [79-81].

The kind of sensors and its location in the network also must be contemplated. In reference [57,58], any kind of optical sensors can be multiplexed in the network which confers an extra degree of freedom in its design. Thus, it can be adapted to every application: distributing the sensors at critical points and even combining different kind of sensors. In most of the developed remote sensor schemes, however, the chosen sensors are FBGs because of its appealing features [41,43,68,69]. The FBGs disposal within the systems is usually in series or in parallel. The first one is a good option when the network is not based on a multiwavelength fiber laser [62,69] because, in this case, achieving power equalization for all the channels is not an easy task which restricts severely the number of multiplexed FBGs [39,59]. This serial topology allows utilizing the power efficiently but the performance of one sensor depends directly on the previous ones. Parallel arrangement, on the other hand, is more suitable for systems based on multiwavelength fiber lasers if channel equalization is considered [66]. Each behavior sensor is independent from the others, in consequence, it makes easier the development of self-healing networks, enables an easy repair of the sensors when needed or exchange the kind of sensor depending on the needs. On the other hand, the power requirements of parallel disposition are much higher.

One of the most restricting factors in the remote sensor systems is the accumulated and amplified Rayleigh scattering. Unlike other broadband noise like ASE, Rayleigh scattering is centered at the same wavelengths as the signal. For this reason, avoiding this noise or distinguishing the contribution of the noise and the signal are complex tasks. Till now, two different ways have been presented. The first one uses an heterodyne detection system [43,68] which relegates the Rayleigh noise to low frequencies and the signal is transferred to noise-free RF frequencies. The second one includes a high-speed pulse-driven swept light source, which is turned off immediately after wavelength sweeping finishes and so the Rayleigh noise is reduced [44,62].

Multiplexing or self-healing capability are other important issues to address as well. In real applications of remote sensor systems, at least, tens of sensors are needed to control the whole structure under survey because either they usually have huge dimensions or the parameters to be assessed are several. In consequence, apart from reducing the cost and complex of the networks, multiplexing capability helps to have an adequate evaluation of all key parameters. Moreover, resilient remote sensor systems must concentrate the attention. Till now, we dare to say that only one scheme has been proposed combining long distance and resilient [66]. Resilience is the ability of continue operating despite one or more points of failure on the network, which will be the key issue for practical FBG sensor systems. The continued operation of the sensor network after accidental or malicious damage is of increasing importance when the structure being monitored is of high value; human safety is at risk or perimeter security is a [4]. Much work must be dedicated to this point.

At all events, no combination of the aforementioned aspects is the answer for all the applications, but there is indeed enough flexibility in order to reach an optimum scheme for any application. To finish, we would like to point out that the foremost aspect to considerer in the design of real remote sensor systems is the economic conditions. Thus, simplicity and cost always must be in mind.

## 4. Conclusions

Multi point sensing fiber optic networks for remote sensor systems are a promising technology with a wide field of practical applications but with some challenges to face. This overview shows the great evolution of these systems in recent years. The most representative systems are explained in ascending order of the system length: beginning with the shortest 22 km and 50 km to finish with the longest till now of 250 km. Along the discussion, the foremost factors on such matter, as kind of amplification, network topology, pump efficiency, type of sensors, noise to overcome, multiplexing and self-healing capability, simplicity and cost are taking into consideration to evaluate each system.

## Figures and Tables

**Figure 11.**
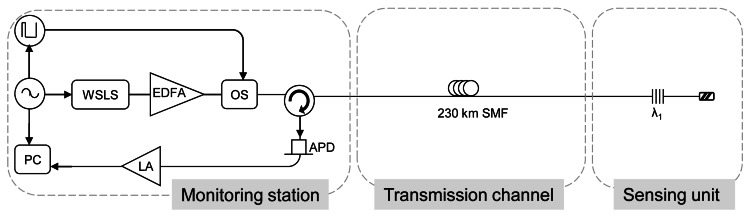
Experimental set-up for the FBG sensor system. APD: avalanche photodetector. L.A.: logarithmic amplifier. WSLS: wavelength-swept light source. OS: Optical switch. Adapted from [10].

**Table 1.** State of the art of remote fiber-optic sensor systems.

**Year/Ref.**	**Amplification Type**	**Network Topology**	**Network Length**	**Sensors Multiplexed**	**SNR**
2003/[56]	Raman	Bus	50 km	1	15 dB
2004/[40]	Raman	Bus	25 km	2	50 dB
2004/[42]	Raman+EDFA	Bus	50 km	1	11 dB
2005/[57]	Raman	Bus	22 km	4	15 dB
2005/[58]	Raman	Bus	35 km	8	27 dB
2005/[59]	Raman	Bus	50 km	1	50 dB
2005/[39]	Raman	Bus	50 km	2	50 dB
2005/[60]	EDWA+SOA	Bus	25 km	3	50 dB
2006/[61]	Raman	Bus	16.5 km	4	16 dB
2006/[11]	Raman+EDFA	Bus/Star	50 km	2	60 dB
2007/[62]	No amplification	Bus	120 km	1	24 dB
2008/[63]	EDFA+SOA	Bus	20 km	3	25 dB
2008/[10]	EDFA	Bus	230 km	1	4 dB
2009/[64]	EDFA	Ring/star	50 km	4	58 dB
2009/[65]	Raman	Bus	50 km	2	50 dB
2010/[66,67]	Raman	Bus/Star	50 km	4	46 dB
2010/[41]	Raman+EDFA	Bus	100 km	1	30 dB
2011/[68]	Raman+Brillouin	Bus	100 km	4	30 dB
2011/[43]	Raman+EDFA+Brillouin	Bus	155 km	2	10 dB
2011/[69]	Raman	Bus	200 km	4	22 dB
2011/[69]	Raman	Bus	250 km	4	18 dB
2011/[44]	No amplification	Bus	253 km	1	3 dB
2011/[70]	Raman	Bus	75 km	2	17 dB
2012/[71]	EDFA	Ring/Star	50 km	2	25 dB
2012/[72]	Raman+EDFA	Bus	150 km	3	1 dB

## References

[1] Akyildiz I.F., Su W., SankarasubRamaniam Y., Cayirci E. (2002). A survey on sensor networks. IEEE Commun. Mag..

[2] Santos J.L., Frazãoa O., Baptista J.M., Jorge P.A.S., Dias I., Araújo F.M., Ferreira L.A. Optical Fibre Sensing Networks.

[3] López O.G., Schires K., Urquhart P., Gueyne N., Duhamel B. (2009). Optical fiber bus protection network to multiplex sensors: Amplification by remotely pumped EDFAs. IEEE Trans. Instrum. Meas..

[4] Li H., Li D., Song G. (2004). Recent applications of fiber optic sensors to health monitoring in civil engineering. Eng. Struct..

[5] Majumder M., Gangopadhyay T.K., Chakraborty A.K., Dasgupta K., Bhattacharya D.K. (2008). Fibre bragg gratings in structural health monitoring-present status and applications. Sens. Actuat. A Phys..

[6] Dandridge A., Kirkendall C., Lopez-Higuera J.M. (2002). Passive Fiber Optic Sensor Networks. Optical Fibre Sensing Technology.

[7] Udd E., Yin S., Ruffin P.B., Yu F.T.S. (2008). Overview Fiber Optic Sensors. Book Fiber Optic Sensors.

[8] Urquhart P., Palezi H., Jardin P. (2011). Optical Fiber Bus Protection Network to Multiplex Sensors: Self-Diagnostic Operation. J. Lightw. Technol..

[9] Mehrani E., Ayoub A., Ayoub A. (2009). Evaluation of Fiber Optic Sensors for Remote Health Monitoring of Bridge Structures. Mater. Struct./Mater. Construct.

[10] Saitoh T., Nakamura K., Takahashi Y., Iida H., Iki Y., Miyagi K. (2008). Ultra-long-distance (230 km) FBG sensor system. Proc. SPIE..

[11] Rao Y., Ran Z., Chen R. (2006). Long-distance fiber bragg grating sensor system with a high optical signal-to-noise ratio based on a tunable fiber ring laser configuration. Opt. Lett..

[12] Guru Prasad A.S., Asokan S., Tatavarti R. Detection of Tsunami Wave Generation and Propagation using Fiber Bragg Grating Sensors.

[13] Nakstad H., Kringlebotn J.T. (2008). Realisation of a full-scale fibre optic ocean bottom seismic system. Proc. SPIE..

[14] Ferraro P., De Natale G. (2002). On the possible use of optical fiber bragg gratings as strain sensors for geodynamical monitoring. Opt. Lasers in Eng..

[15] Wei C., Lai C., Liu S., Chung W.H., Ho T.K., Tam H., Ho S.L., McCusker A., Kam J., Lee K.Y. (2010). A fiber bragg grating sensor system for train axle counting. IEEE Sens. J.

[16] Zornoza A., Olier D., Sagues M., Loayssa A. (2010). Brillouin distributed sensor using RF shaping of pump pulses. Meas. Sci. Technol..

[17] Rodríguez-Barrios F., Martín-López S., Carrasco-Sanz A., Corredera P., Ania-Castañón J.D., Thévenaz L., González-Herráez M. (2010). Distributed Brillouin Fiber Sensor Assisted by First-Order Raman Amplification. J. Lightw. Technol..

[18] Song K.Y., Chin S., Primerov N., Thevenaz L. (2010). Time-domain distributed fiber sensor with 1 cm spatial resolution based on brillouin dynamic grating. J. Lightw. Technol..

[19] Soto M.A., Bolognini G., Di Pasquale F. (2011). Optimization of long-range BOTDA sensors with high resolution using first-order bi-directional Raman amplification. Opt. Express.

[20] Soto M.A., Faralli S., Taki M., Bolognini G., Di Pasquale F. (2011). BOTDA sensor with 2-m spatial resolution over 120 km distance using bi-directional distributed Raman amplification. Proc. SPIE..

[21] Angulo-Vinuesa X., Martin-Lopez S., Nuno J., Corredera P., Ania-Castanon J.D., Thévenaz L., Gonzalez-Herraez M. (2011). Hot spot detection over 100 km with 2 meter resolution in a Raman-assisted brillouin distributed sensor. Proc. SPIE..

[22] Dong Y., Bao X., Chen L. (2011). 100-km Sensing range brillouin optical time domain analysis based on time-division multiplexing. Proc. SPIE..

[23] Rao Y. (2006). Recent progress in fiber-optic extrinsic fabry-perot interferometric sensors. Opt. Fiber Technol..

[24] Tuck C.J., Hague R., Doyle C. (2006). Low cost optical fibre based fabry-perot strain sensor production. Meas. Sci. Technol..

[25] Pinet É. (2009). Fabry-pérot fiber-optic sensors for physical parameters measurement in challenging conditions. J. Sens..

[26] Taylor H.R., Yin S., Ruffin P.B., Yu F.T.S. (2008). Fiber Optic Sensors Based upon the Fabry-Perot Interferometer. Fiber Optic Sensors.

[27] Chow J.H., Littler I.C.M., McClelland D.E., Gray M.B. (2006). Long distance, high performance remote strain sensing with a fiber fabry-perot by radio-frequency laser modulation. Proc SPIE..

[28] Chow J.H., Littler I.C.M., McClelland D.E., Gray M.B. A 100 km Ultra-High Performance Fiber Sensing System.

[29] Kersey A.D., Davis M.A., Patrick H.J., LeBlanc M., Koo K.P., Askins C.G., Putnam M.A., Friebele E.J. (1997). Fiber grating sensors. J. Lightw. Technol..

[30] Vohra S.T., Lopez-Higuera J.M. (2002). Optical Fibre Gratings Applications. Optical Fibre Sensing Technoloy.

[31] Zhao Y., Liao Y. (2004). Discrimination methods and demodulation techniques for fiber bragg grating sensors. Opt. Lasers Eng..

[32] Jones J.D.C., MacPherson W.N., Lopez-Higuera J.M (2002). Discrimination Techniques for Optical Sensors. Optical Fibre Sensing Technology.

[33] Lee B., Jeong Y., Yin S., Ruffin P.B., Yu F.T.S. (2008). Interrogation Techniques for Fiber Grating Sensors and the Theory of Fiber Gratings. Fiber Optic Sensors.

[34] Santos J.L., MacPherson W.N., Lopez-Higuera J.M. (2002). Fiber Bragg Grating Interrogation Techniques. Optical Fibre Sensing Technoloy.

[35] Guo H., Xiao G., Mrad N., Yao J. (2011). Fiber optic sensors for structural health monitoring of air platforms. Sensors.

[36] Rodrigues C., Félix C., Lage A., Figueiras J. (2010). Development of a long-term monitoring system based on FBG sensors applied to concrete bridges. Eng. Struct..

[37] Filograno M.L., Corredera Guillén P., Rodríguez-Barrios A., Martin-López S., Rodríguez-Plaza M., Andrés-Alguacil Á., González-Herráez M. (2012). Real-Time Monitoring of Railway Traffic using Fiber Bragg Grating Sensors. IEEE Sens. J.

[38] Diaz S., Abad S., Lopez-Amo M. (2008). Fiber-optic sensor active networking with distributed erbium-doped fiber and Raman amplification. Laser Photonics Rev..

[39] Han Y., Tran T.V.A., Kim S., Lee S.B. (2005). Multiwavelength Raman-fiber-laser-based long-distance remote sensor for simultaneous measurement of strain and temperature. Opt. Lett..

[40] Peng P., Tseng H., Chi S. (2004). Long-distance FBG sensor system using a linear-cavity fiber Raman laser scheme. IEEE Photonics Technol. Lett..

[41] Hu J., Chen Z., Yang X., Ng J., Yu C. (2010). 100-km Long distance fiber bragg grating sensor system based on erbium-doped fiber and Raman amplification. IEEE Photonics Technol. Lett..

[42] Lee J.H., Chang Y.M., Han Y., Chung H., Kim S.H., Lee S.B. (2004). Raman amplifier-based long-distance remote, strain and temperature sensing system using an erbium-doped fiber and a fiber Bragg grating. Opt. Express.

[43] Leandro D., Ullan A., Lopez-Amo M., Lopez-Higuera J.M., Loayssa A. (2011). Remote (155 km) fiber Bragg grating interrogation technique combining Raman, brillouin and erbium gain in a fiber laser. IEEE Photonics Techno. Lett..

[44] Bravo M., Baptista J.M., Santos J.L., Lopez-Amo M., Frazão O. (2011). Ultralong 250 km remote sensor system based on a fiber loop mirror interrogated by an optical time-domain reflectometer. Opt. Lett..

[45] Rottwitt K., Headley C., Agrawal G.P. (2005). Distributed Raman Amplifiers. Raman Amplification in Fiber Optical Communication Systems.

[46] Stolen R.H., Islam M.N. (2004). Fundamentals of Raman Amplification in Fibers. Raman Amplifiers for Telecommunication 1, Physical Principle.

[47] Bromage J. (2004). Raman amplification for fiber communications systems. J. Lightw. Technol..

[48] Islam M.N., Islam M.N. (2004). Raman Amplification in Telecommunications. Raman Amplifiers for Telecommunication 1, Physical Principle.

[49] Sheng Z. (2005). Theoretical analysis of rayleigh backscattering noise in fiber Raman amplifiers. Commun. Theor. Phys..

[50] Hansen P.B., Eskildsen L., Stentz A.J., Strasser T.A., Judkins J., DeMarco J.J., Pedrazzani R., DiGiovanni D.J. (1998). Rayleigh Scattering limitations in distributed Raman pre-amplifiers. IEEE Photonics Technol. Lett..

[51] Fludger C.R.S., Mears R.J. (2001). Electrical measurements of multipath interference in distributed Raman amplifiers. J. Lightw. Technol..

[52] Bromage J., Winzer P.J., Essiambre R.J., Islam M.N. (2004). Multiple Path Interference and its Impact on System Design. Raman Amplifiers for Telecommunication 2, Sub-Systems and Systems.

[53] Lewis S.A.E., Chernikov S.V., Taylor J.R. (2000). Characterization of double rayleigh scatter noise in Raman amplifiers. IEEE Photonics Technol. Lett..

[54] Kim S., Kwon J., Kim S., Lee B. (2001). Multiplexed strain sensor using fiber grating-tuned fiber laser with a semiconductor optical amplifier. IEEE Photonics Technol. Lett..

[55] May-Alarcón M., Kuzin E.A., Vázquez-Sánchez R.A., Shlyagin M.G., Marquez-Borbón I. (2003). Multipoint fiber Bragg grating laser sensor interrogated by the intermodal beating frequency. Opt. Eng..

[56] Nakajima Y., Shindo Y., Yoshikawa T. (2003). Novel concept as long-distance transmission FBG sensor system using distributed Raman amplifier. Proc. SPIE..

[57] Díaz S., Lasheras G., López-Amo M., Urquhart P., Jáuregui C., López-Higuera J.M. (2006). Wavelength-division-multiplexed distributed fiber Raman amplifier bus network for sensors. Proc. SPIE..

[58] Diaz S., Lasheras G., Lopez-Amo M. (2005). WDM bi-directional transmission over 35 km amplified fiber-optic bus network using Raman amplification for optical sensors. Opt. Express.

[59] Han Y., Tran T.V.A., Kim S., Lep S.B. (2005). Development of a multiwavelength Raman fiber laser based on phase-shifted fiber bragg gratings for long-distance remote-sensing applications. Opt. Lett..

[60] Peng P., Feng K., Peng W., Chiou H., Chang C., Chi S. (2005). Long-distance fiber grating sensor system using a fiber ring laser with EDWA and SOA. Opt. Commun..

[61] Diaz S., Lopez-Amo M. (2006). Comparison of wavelength-division-multiplexed distributed fiber Raman amplifier networks for sensors. Opt. Express.

[62] Saitoh T., Nakamura K., Takahashi Y., Iida H., Iki Y., Miyagi K. (2007). Ultra-long-distance fiber Bragg grating sensor system. IEEE Photonics Technol. Lett..

[63] Daru C., Chester S., Sailing H. (2008). Multiple fiber Bragg grating interrogation based on a spectrum-limited fourier domain mode-locking fiber laser. Opt. Lett..

[64] Perez-Herrera R.A., Diaz S., Fernández-Vallejo M., López-Amo M., Quintela M.A., Lopez-Higuera J.M. (2009). Switchable multi-wavelength erbium-doped fiber laser for remote sensing. Proc. SPIE..

[65] Han Y. (2009). Long-distance Remote sensors for simultaneous measurement of strain and temperature based on multiwavelength fiber lasers. Proc. SPIE..

[66] Fernandez-Vallejo M., Díaz S., Perez-Herrera R.A., Passaro D., Selleri S., Quintela M.A., López Higuera J.M., Lopez-Amo M. (2010). Resilient long-distance sensor system using a multiwavelength Raman laser. Meas. Sci. Technol..

[67] Fernandez-Vallejo M., Díaz S., Perez-Herrera R.A., Passaro D., Selleri S., Quintela M.A., López Higuera J.M., Lopez-Amo M. (2009). Resilient long-distance sensor system using a multiwavelength Raman laser. Proc. SPIE..

[68] Fernandez-Vallejo M., Leandro D., Loayssa A., Lopez-Amo M. (2011). Fiber Bragg grating interrogation technique for remote sensing (100 km) using a hybrid brillouin-Raman fiber laser. Proc. SPIE..

[69] Fernandez-Vallejo M., Rota-Rodrigo S., Lopez-Amo M. (2011). Remote (250 km) fiber Bragg grating multiplexing system. Sensors.

[70] Han Y.G. (2011). A long-distance remote sensing technique using a multiwavelength Raman fiber laser based on fiber Bragg gratings embedded in a quartz tube. IEEE Sens. J.

[71] Bravo M., Fernández Vallejo M., Lopez-Amo M. (2012). Hybrid OTDR-fiber laser system for remote sensor multiplexing. IEEE Sens. J.

[72] Hu J., Chen Z., Yu C. (2011). 150-km long distance FBG temperature and vibration sensor system based on stimulated Raman amplification. IEEE J. Lightw. Technol..

[73] Lopez-Amo M. (2006). Abad S. Amplified fiberoptic networks for sensor multiplexing. Jpn. J. Appl. Phys..

[74] Lopez-Izquierdo E., Urquhart P., Lopez-Amo M. (2007). Protection architectures for WDM optical fibre bus sensor arrays. J. Eng..

[75] Carena A., Curri V., Poggiolini P. (2001). On the optimization of hybrid Raman/erbium-doped fiber amplifiers. IEEE Photonics Technol. Lett..

[76] Jenkins R.B., Sova R.M., Joseph R.I. (2007). Steady-state noise analysis of spontaneous and stimulated brillouin scattering in optical fibers. J. Lightw. Technol..

[77] Chraplyvy A.R. (1990). Limitations on lightwave communications imposed by optical-fiber nonlinearities. J. Lightw. Technol..

[78] Pérez-Herrera R.A., Quintela M.A., Fernández-Vallejo M., Quintela A., López-Amo M., López-Higuera J.M. (2009). Stability comparison of two ring resonator structures for multiwavelength fiber lasers using highly doped er-fibers. J. Lightw. Technol..

[79] Montoya V., López-Amo M., Abad S. (2000). Improved double-fiber-bus with distributed optical amplification for wavelength-division multiplexing of photonic sensors. IEEE Photonics Technol. Lett..

[80] Diaz S., Cerrolaza B., Lasheras G., Lopez-Amo M. (2007). Double Raman amplified bus networks for wavelength-division multiplexing of fiber-optic sensors. J. Lightw. Technol..

[81] Digonnet M.J.F., Vakoc B.J., Hodgson C.W., Kino G.S. (2004). Acoustic fiber sensor arrays. Proc. SPIE..

